# Premature Termination of MexR Leads to Overexpression of MexAB-OprM Efflux Pump in *Pseudomonas aeruginosa* in a Tertiary Referral Hospital in India

**DOI:** 10.1371/journal.pone.0149156

**Published:** 2016-02-11

**Authors:** Debarati Choudhury, Anamika Ghosh, Debadatta Dhar Chanda, Anupam Das Talukdar, Manabendra Dutta Choudhury, Deepjyoti Paul, Anand Prakash Maurya, Atanu Chakravorty, Amitabha Bhattacharjee

**Affiliations:** 1 Department of Life Science & Bioinformatics, Assam University, Silchar, Assam, India; 2 Department of Microbiology, Assam University, Silchar, Assam, India; 3 Department of Microbiology, Silchar Medical College and Hospital, Silchar, Assam, India; Naval Research Laboratory, UNITED STATES

## Abstract

**Objectives:**

The present study was undertaken to investigate the mutations that are present in *mexR* gene of multidrug resistant (MDR) isolates of *Pseudomonas aeruginosa* collected from a tertiary referral hospital of north east India.

**Methods:**

76 MDR clinical isolates of *P*. *aeruginosa* were obtained from the patients who were admitted to or attended the clinics of Silchar medical college and hospital. They were screened phenotypically for the presence of efflux pump activity by an inhibitor based method. Acquired resistance mechanisms were detected by multiplex PCR. Real time PCR was performed to study the expression of *mexA* gene of MexAB-OprM efflux pump in isolates with increase efflux pump activity. *mexR* gene of the isolates with overexpressed MexAB-OprM efflux pump was amplified, sequenced and analysed.

**Results:**

Out of 76 MDR isolates, 24 were found to exhibit efflux pump activity phenotypically against ciprofloxacin and meropenem. Acquired resistance mechanisms were absent in 11 of them and among those isolates, 8 of them overexpressed MexAB-OprM. All the 8 isolates possessed mutation in *mexR* gene. 11 transversions, 4 transitions, 2 deletion mutations and 2 insertion mutations were found in all the isolates. However, the most significant observation was the formation of a termination codon at 35^th^ position which resulted in the termination of the polypeptide and leads to overexpression of the MexAB-OprM efflux pump.

**Conclusions:**

This study highlighted emergence of a novel mutation which is probably associated with multi drug resistance. Therefore, further investigations and actions are needed to prevent or at least hold back the expansion and emergence of newer mutations in nosocomial pathogens which may compromise future treatment options.

## Introduction

*Pseudomonas aeruginosa* is one of the leading opportunistic pathogen and is often found to be associated with nosocomial infections mainly in immunocompromised patients [1]. Infections caused by this organism are hard to treat because of the intrinsic resistance and the outstanding ability to acquire resistance mechanisms to several classes of antimicrobial agents during therapy which ultimately results into treatment failure. Development of resistance after exposure to antibiotics and subsequent cross-resistance between agents confers multidrug-resistant (MDR) phenotype in *P*. *aeruginosa* [2]. In absence of acquired resistance determinants, multidrug resistance (MDR) is mediated by various intrinsic resistance mechanisms which include lower outer membrane permeability and increased activity of drug specific efflux pumps with wide substrate profiles [3].

Clinically significant efflux pumps of *P*. *aeruginosa* belong to resistance-nodulation-cell division (RND) family [3]. Of all the RND pumps of *P*. *aeruginosa*, MexAB-OprM has the most wide substrate profile and can pump out a number of antibiotics belonging to different classes including beta-lactam (carboxypenicillins, aztreonam, extended-spectrum cephalosporins, penems, the carbapenems such as meropenem and panipenem except imipenem and biapenem); fluoroquinolones; tetracyclines; chloramphenicol; macrolides; novobiocin; trimethoprin and sulfonamides. It is constitutively expressed in wild type cells and overexpression of this particular pump can lead to multidrug resistant phenotype [4].

One of the important regulatory gene of MexAB-OprM operon is *mexR* which is located upstream of the MexAB-OprM and encodes a repressor MexR that binds as a homodimer between the intergenic region of *mexR* and *mexA* resulting in transcriptional repression of MexAB-OprM and negative autoregulation of its expression [5]. Mutations in *mexR* (*nalB* mutants) lead to hyperexpression of MexAB-OprM. In case of *nalB* mutants, mutations (insertion, deletion, substitution or insertion of IS elements) in *mexR* leads to expression of a defective MexR protein which cannot act as repressor because of loss of its dimerization/ DNA binding ability. *nalC* type mutants overexpress MexAB-OprM at a lower level than *nalB* mutatnts. *nalC* type mutations are found within the PA3721 gene (*nalC* gene). The repressor NalC encoded by *nalC* gene negatively autoregulates PA3720-PA3719 operon. Mutations in *nalC* result in loss of NalC repressor that causes overexpression of PA3720-PA3719. PA3719 (renamed ArmR) ultimately interacts with MexR and causes upregulation of *mexAB*-*oprM* [3]. Another repressor of the MexAB-OprM operon, NalD is encoded by a gene *nalD*. It binds to a sequence downstream of *mexR* and upstream of *mexAB-OprM*. NalD represses expression of *mexA* by overlapping the proximal promoter of *mexA* [6–7]. Apart from mutation in regulatory gene, oxidative stress was also found to increase transcription of MexAB-OprM. Oxidation distorts the conformation of MexR that cannot bind to the *mexR-mexA* intergenic region which result in hyperexpression [3].

The rate of antimicrobial resistance in northeastern part of India is quite high [8] and data regarding the mutation pattern in *mexR* gene is lacking from this part of the world. This present study was undertaken with a view to examine whether the mutations from this region are same as existing knowledge or any new mutation is prevailing in this region. The objective of the current study was to investigate the mutations that are present in *mexR* genes of MDR isolates collected from a tertiary referral hospital of north east India.

## Materials and Methods

### Bacterial Isolates

A total of 76 MDR clinical isolates of *P*. *aeruginosa* were obtained from the patients who were admitted to or attended the clinics of Silchar medical college and hospital, Assam, India from March, 2014 to August, 2014. MDR *P*. *aeruginosa* were selected on the basis of non-susceptibility to at least one agent in at least 3 antimicrobial classes of the following: cephalosporin (ceftazidime), β-lactam/ β-lactamase inhibitor combination, (piperacillin/tazobactam), carbapenems (imipenem, meropenem), fluoroquinolones (ciprofloxacin), aminoglycosides (gentamicin, amikacin) [9]. *P*. *aeruginosa* PAO1 (Kindly donated by Prof. Keith Poole, Queens University, Canada) was taken as the quality control strain.

### Ethical Approval

The work was approved by Institutional Ethical committee of Assam University, Silchar with reference no.: IEC/AUS/2013-003. The authors confirmed that written consent in a prescribe format was obtained from patient. Consent process was approved by the institutional ethics committee.

### Antimicrobial susceptibility testing and Minimum Inhibitory Concentration determination

Antibiotic susceptibility testing was performed on Mueller Hinton agar (Himedia, Mumbai, India) plates by Kirby-Bauer disc diffusion method and interpreted as per CLSI recommendations [10]. The antibiotics tested viz., meropenem (10μg) ciprofloxacin (5μg), amikacin (30μg), gentamicin (10μg), carbenicillin (10μg), polymixin B (300 μg), ceftazidime (30 μg), Piperacillin-Tazobactam (100/10 μg), faropenem (5μg) (Himedia, Mumbai, India).

Minimum inhibitory concentration (MIC) was determined on Muller Hinton Agar plates by agar dilution method against meropenem and ciprofloxacin (Himedia, Mumbai, India) according to CLSI guidelines and interpreted according to CLSI breakpoint [10].

### Assessment of efflux pump activity using inhibitor based method

Efflux pump activity of the strains were phenotypically detected by using meropenem (10 mg, Himedia, Mumbai) and ciprofloxacin (5 mg, Himedia, Mumbai) with and without CCCP (carbonyl cyanide m-chlorophenylhydrazone, 100mM, Himedia, Mumbai) [11].

MIC reduction assay was performed with meropenem and ciprofloxacin alone and in combination with CCCP at a concentration of 12.5 μM [11, 12]. An efflux pump-overexpressed phenotype was defined as any strain exhibiting at least a twofold decrease in MIC when tested in the presence of CCCP.

### Detection of acquired resistance determinants

To confirm the absence of specific carbapenemase and fluroquinolone resistance determinants, PCR assay was performed for detection of *bla*_VIM_, *bla*_NDM_, *bla*_IMP_, *bla*_OXA-48_, *bla*_OXA_-_23, -24/40, -58,_ *qnrA*, *qnrB*, *qnrS*, and *qepA*. The reaction conditions and primers were used as described previously [13–17].

### Detection of transcription of *mexA* and *OprD* gene by quantitative real time PCR

Expression of *mexA* and *OprD* gene was determined by qRT-PCR as described previously with some modifications [18, 19]. RNA was isolated from culture grown to log phase of growth using RNeasy Kit (Qiagen, Hilden, Germany). cDNA was prepared from mRNA using QuantiTect Reverse Transcription Kit (Qiagen, Hilden, Germany). All the Preapared cDNA were subjected to semiquantitative PCR. mRNA transcription level was determined using SYBR^®^ Green PCR Master Mix (Applied Biosystems, Foster City, CA) in Step one plus real time PCR system (Applied Biosystem, USA). Relative quantities of gene expression were calculated using the ΔΔCt method. Expression of the 30S ribosomal gene *rpsL* was determined in parallel as internal control.

Samples were run in triplicate. 50 ng of RNA which was reverse transcribed, was used as template. MexAB-OprM efflux pump was considered overexpressed when transcription level was atleast 2-fold higher compared with that of *P*. *aeruginosa* PAO1 [20]. Reduced OprD expression was considered significant when it was ≤30% compared with that of *P*. *aeruginosa* PAO1 [19].

### Detection of mutation in *mexR* gene

The *mexR* gene of MDR isolates which do not possess acquired resistance determinants along with *P*. *aeruginosa* PAO1 were amplified using *mexR* gene-specific primers (forward primer: 5’-TCAGAACCTGAAACAAGGTTG-3’ and reverse primer: 5’- ATCGCCGGCGTTTTTCATTGTG-3’) (This study). Each single reaction mixture (25μl) contained 4μl of template DNA (approx. 100 ng/μl), 1μl of each primer (10 picomole), 12.5μl of GoTaq green Master Mix 2X DNA polymerase (Promega, Madison, USA) and nuclease free water.

The reaction condition was 3 min at 95°C; 35 cycles of 25 s at 95°C, 1 min at 52°C and 2 min at 72°C, and finally, 10 min at 72°C. Reaction was run in Applied Biosystem Veriti^®^ thermal cycler (Applied Biosystem, USA). The amplified products were analyzed by 1% (w/w) agarose gel electrophoresis and visualized after staining with ethidium bromide. They were sequenced, analyzed, and compared with that of the reference strain *P*. *aeruginosa* PAO1 [7]. 30μl of purified PCR products were used for sequencing along with 20μl of primers each (20 picomole each primers). Sequencing was done by Xcelaris Lab, Ahmedabad, India and sequence results were analyzed using BLAST suite program of NCBI (http://blast.ncbi.nlm.nih.gov).

### Cloning of *mexR* gene in a *ΔmexR* strain and analysis of its effect on expression of MexAB-OprM

The mutated *mexR* gene from AM-D-529 and wildtype *mexR* gene from *P*. *aeruginosa* PAO1 were amplified using forward primer 5’AGTCTTGGACTTTGTTCCAAC3’ and reverse primer: 5’ TAGGGTCGGCGTTCTTGCATGG 3’. PCR amplification was performed using 50 μl of total reaction volume. Reactions were run under the following conditions: initial denaturation at 94°C for 2 min, 32 cycles of 94°C for 25 Sec., 52°C for 1min, 72°C for 3min and final extension at 72°C for 10 min. PCR products were examined on 1.0% (w/v) agarose gels and purified using the GeneJET Gel extraction Kit (Thermo Scientific). Amplified products were cloned into pET-30a vector (Novagen) between NdeI and XhoI sites and transformed in K767-PAO1 Δ*mexR* (Kindly donated by Prof. K. Poole of Queens University, Canada) by heat shock method.

Expression of *mexA* gene of transformant was determined by real time PCR as described above.

MIC of transformants against ciprofloxacin and meropenem was determined.

### DNA fingerprinting by REP PCR

REP PCR was performed to determine clonal relatedness of all the isolates selected under the present work. The primers and reaction condition used were same as described previously [21].

## Results

The antibiogram profile of MDR isolates are listed in Table 1. The study isolates were of multidrug resistant phenotype. Polymixin B was found to have moderate activity. Other group of antibiotics came up with low efficacy.

**Table 1:** Antibiogram profile of MDR *P*. *aeruginosa*.

Antibiotics	Total no of samples	Total no of susceptible samples	% of susceptible isolates
Amikacin	76	11	14.47
Gentamycin	76	13	17.1
Pipericillin-tazobactum	76	10	13.15
Faropenem	76	9	11.84
Polymixin b	76	25	32.89
Carbenicillin	76	7	9.21
Ceftazidime	76	9	11.84
Meropenem	76	12	15.78
Ciprofloxacin	76	16	21.05

Among 76 MDR isolates 31 and 28 were found to exhibit enhanced efflux pump activity against meropenem and ciprofloxacin respectively. 24 isolates demonstrated efflux pump activity against both meropenem and ciprofloxacin. They were above breakpoint level for meropenem and ciprofloxacin a sharp reduction in MIC was observed in all the isolates when CCCP was added (Table 2). Multiplex PCR could not establish the presence of targeted acquired resistant determinants in 11 of them.

**Table 2:** MIC reduction assay of isolates that demonstrated synergy between CCCP and one of the tested antibiotics (meropenem and ciprofloxacin).

Serial No	Isolate Identification No	MIC against Meropenem (μg/ml)	MIC against Meropenem (after addition of CCCP) (μg/ml)	MIC against Ciprofloxacin (μg/ml)	MIC against Ciprofloxacin (after addition of CCCP) (μg/ml)
1	AM-D-64	32	16	16	8
2	AM-D-335	64	16	32	16
3	AM-D-326	32	16	16	8
4	AM-D-352	32	8	32	16
5	AM-D-173	32	16	16	4
6	AM-D-529	64	16	32	4
7	AM-D-75	64	32	32	16
8	AM-D-608	64	16	64	32
9	AM-D-131	64	16	64	8
10	AM-D-146	32	8	32	4
11	AM-D-67	64	16	32	8
12	AM-D-466	64	32	64	16
13	AM-D-414	128	64	64	16
14	AM-D-329	32	16	16	4
15	AM-D-219	128	64	64	8
16	AM-D-135	64	32	32	8
17	AM-D-525	128	64	128	32
18	AM-D-288	128	32	64	32
19	AM-D-420	64	32	16	4
20	AM-D-579	64	16	32	8
21	AM-D-238	32	8	16	8
22	AM-D-139	128	64	128	64
23	AM-D-571	64	32	32	16
24	AM-D-609	64	8	32	8
25	PAO1	0.5	0.5	1	1

As these 11 isolates were devoid of acquired resistance genes, so expression of MexAB-OprM efflux pump and *OprD* was investigated in 11 isolates that demonstrated efflux pump activity phenotypically. Studies revealed that 8 isolates overexpressed MexAB-OprM efflux pump and 2 isolates had reduced *OprD* expression (Table 3).

**Table 3:** mRNA expression of *mexA* and *OprD* gene.

Serial no	Isolate Identification no	mRNA expression of *mexA* gene (Mean of triplicates ± standard error) relative to expression of P. aeruginosa PAO1 which is assigned a value 1*	mRNA expression of *OprD* gene (Mean of triplicates ± standard error) relative to expression of P. aeruginosa PAO1 which is assigned a value 1*
1	AM-D-64	2.53^a^±0.41	1.14±0.21
2	AM-D-335	1.83±0.11	0.074^b^±0.02
3	AM-D-326	1.46±0.17	0.097 ^b^ ±0.03
4	AM-D-352	3.03 ^a^ ±0.3	1.73±0.08
5	AM-D-173	4.43 ^a^ ±0.41	0.94±0.16
6	AM-D-529	6.3 ^a^ ±0.61	1.43±0.43
7	AM-D-75	1.79±0.1	0.67±0.16
8	AM-D-608	15 ^a^ ±0.92	0.74±0.22
9	AM-D-131	8.75 ^a^ ±0.38	0.49±0.24
10	AM-D-146	9.4 ^a^ ±0.89	1.24±0.29
11	AM-D-67	6.87 ^a^ ±0.32	1.8±0.11

^a^ Isolates overexpressed mexA gene;

^b^ Isolates with reduced OprD expression; normalization was done by Step one plus software (Applied Biosystems, Foster City, CA)

**Fig 1:**
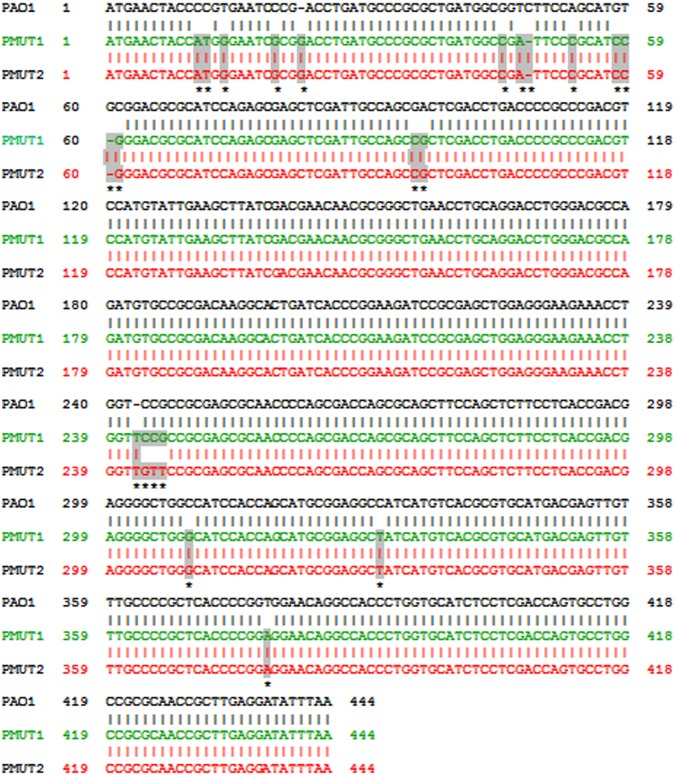
Sequence alignment of the two type of *mexR* sequences (PMUT1 and PMUT2) with the *mexR* sequence of PAO1. PMUT1 sequence corresponds to the first type of mutation pattern found in five isolates (AM-D-64, AM-D-352, AM-D-173, AM-D-529 and AM-D-608). PMUT2 corresponds to the second type of mutation pattern found in three isolates (AM-D-131, AM-D-146, AM-D-67). The mutations have been highlighted in grey colour and were makred with ‘*’.

**Fig 2:**
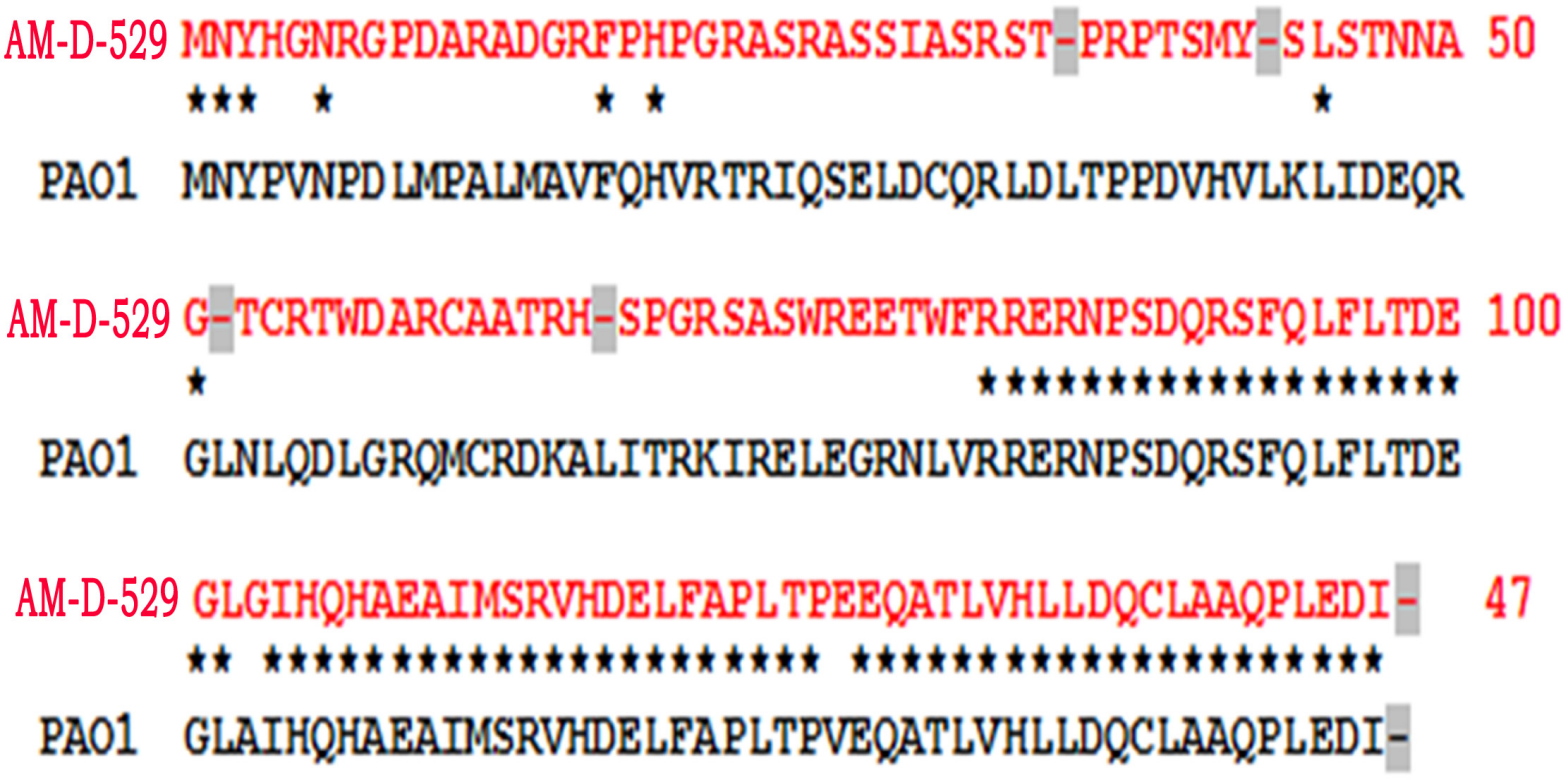
Protein sequence alignment of *mexR* gene of one *P*. *aeruginosa* isolate (AM-D-529, having mutation in *mexR* gene) with *mexR* gene of *P*. *aeruginosa* PAO1. (Similar amino acids were marked as ‘*’. Stop codons are highlighted in grey colour).

In order to determine the contribution of mutation in *mexR* gene in overexpression of MexAB-OprM in the isolates, sequencing of the *mexR* gene was done. A 577 base pair of region of DNA including the *mexR* gene was amplified for all the 11 isolates. Strains that did not overexpress *mexA* had *mexR* sequence identical to that of PAO1 (as published by Poole in reference 7). But all the 8 strains with overexpressed *mexA* gene were found to possess mutation in *mexR*. Five isolates (AM-D-64, AM-D-352, AM-D-173, AM-D-529 and AM-D-608) were found to have 19 point mutations (PMUT1) and the rest 3 isolates carried 22 point mutation (PMUT2) (Fig 1). 11 transversions and 4 transitions were common in all the isolates. Two insertion mutation of a single nucleotide was found between 22^th^ and 23^rd^ base and in between 242^th^ and 243^rd^ base by G and T respectively (starting with position 1 at the A of the start codon of *mexR*). At 48^th^ and 60^th^ base, deletion of single nucleotide was observed in case of all the 8 isolates. Mutations at multiple sites lead to premature termination of the peptide. Termination codon was formed at 35^th^, 43^th^, 52^th^ and 67^th^ position as a result of mutations. Other than that, changes in amino acids were also observed at number of positions (Fig 2). It can be clearly observed that mutations not only caused the premature termination of the peptide but also lead to the formation of an altered peptide. Isolates AM-D-67, AM-D-131 and AM-D-146 in addition to the above mutations also carried three extra mutations; one transition at 245^th^ base and two tranverstions at 244^th^ and 246^th^ base (Fig 1). Two *mexR* gene sequences with PMUT1 and PMUT2 type of mutation pattern have been submitted in GenBank with accession numbers PMUT1 KT026105 and PMUT2 KT026106 respectively.

In order to further support the finding that this novel mutation in *mexR* contributed in overexpression of MexAB-OprM pump, the mutated *mexR* gene and the wildtype *mexR* gene were cloned in a mutant strain that lacked *mexR*. The expression of *mexA* gene in case of the transformant carring the mutated *mexR* gene was found to be approximately 4 fold higher (RQ value of transformant = 4.2) than that of *P*. *aeruginosa* PAO1. Whereas, expression of *mexA* gene in transformant carrying the wildtype *mexR* gene was around in the same level as that of PAO1 (RQ value = 1.48). Transformant with mutated *mexR* was found to be intermediately resistant towards ciprofloxacin (MIC = 2 μg/ml) and susceptible towards meropenem (MIC = 4 μg/ml).

**Fig 3:**
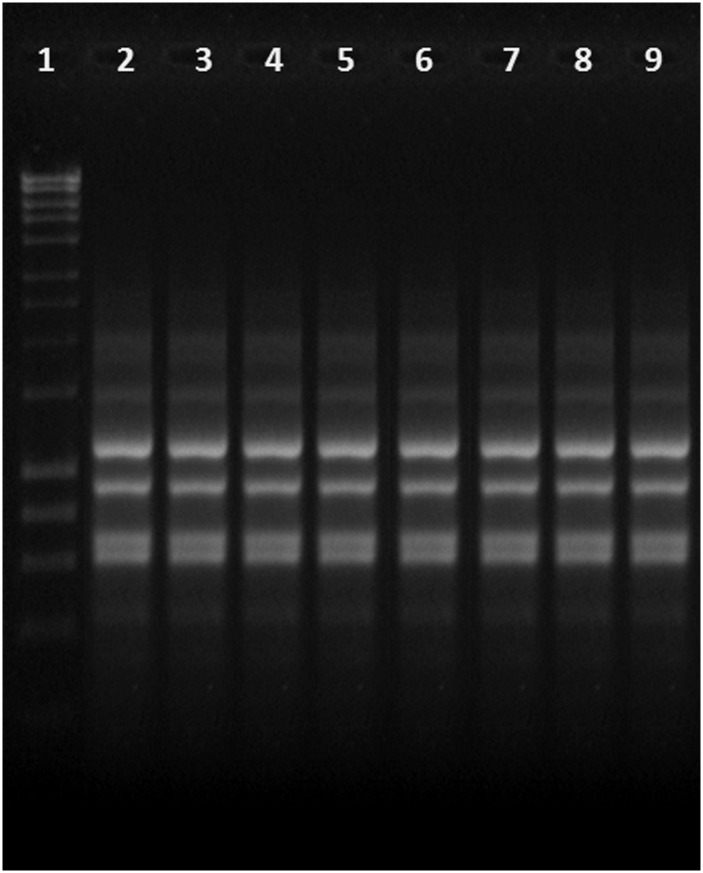
REP PCR showing same REP types of eight *P*. *aeruginosa* isolates with mutated *mexR* gene. Lane 1: Hyper ladder (1 kb); lane 2 to 9: test samples.

All the eight isolates with mutated *mexR* gene were found to be of same REP type (Fig 3).

## Discussion

Efflux pump mediated resistance is posing a great threat to the treatment of infections associated with *P*. *aeruginosa* in hospital settings. Overexpression of MexAB-OprM efflux pump has known to confer multi drug resistance in clinical isolates of *P*. *aeruginosa* [1]. MexR, the product of the regulator gene *mexR* is a transcriptional repressor of the mexA-mexB-oprM efflux pump operon [7]. Mutations in *mexR* leading to overexpression of MexAB-OprM efflux pump has been reported by many authors [7, 20, 22, 23].

In this study, mutation in *mexR* gene of MDR isolates of *P*. *aeruginosa* from a tertiary referral hospital of north east India was investigated. Out of 76 MDR isolates, 11 were found to lack plasmid mediated resistance determinants. As these 11 isolates exhibited efflux pump activity, so expression of MexAB-OprM was investigated in them. It was found that 8 of them overexpressed MexAB-OprM and carried mutation in their *mexR* gene. A unique mutation pattern has been observed in the present study.

Several mutations in *mexR* gene have been reported in previous studies. Ziha-Zafiri *et al*. in 1998 reported several mutations (insertion, deletion and point mutation) in *mexR* genes of post therapy *P*. *aeruginosa* isolates resistant to antipseudomonal beta-lactam antibiotics. They reported a T to A transversion at 377^th^ position which resulted in the substitution of Val to Glu [22]. In the present study, this particular point mutation was observed. They also reported two amino acids substitution at 8^th^ and 66^th^ codons (Asp8Glu and Ala66Val). Though in the present study, substitutions were found at the same position but in this case Asp8 was substituted by Gly and Ala66 by His. Val126 to Glu substitution observed in the present study had been reported previously by a number of authors [20, 22, 23]. The substitution was considered to be insignificant. Llanes *et al*. in 2004 reported a Lys44 to Met susbstitution [23] but we found Lys44 to be substituted by Ser. In this study, a large number of amino acid substitution was observed in the DNA binding domain (residues 37 to 97) [24] of the MexR peptide (Fig 2).

Contribution of mutation in *mexR* towards overexpression of *mexA* gene was further confirmed by cloning the mutated *mexR* gene in a strain that lack *mexR* gene. *mexA* was found to be overexpressed in the transformant. But this mutation conferred intermediate resistance towards ciprofloxacin with an MIC of 2 μg/ml but could not provide resistance to meropenem. This is however obvious as per existing literature overexpression of MexAB-OprM alone could confer resistance to quinolones but supplementary mechanisms (downregulation of porin OprD) in addition to MexAB-OprM overexpresssion is needed for resistance to meropenem [25].

Though some of the mutations have already been reported by previous studies (amino acid substitution at 8^th^, 44^th^, 66^th^, 126^th^) [20, 22, 23] the most important mutation found in this study was the formation of a stop codon at 35^th^ position that leads to termination of the peptide. The other mutations beyond the termination codon can be considered as insignificant because the peptide had already been terminated. An interesting finding of the study is that a single clone (REP type) of *P*. *aeruginosa* carrying a novel mutation in the regulatory gene (*mexR*) which has not been reported earlier was responsible for expansion in the hospital environment and contributed overexpression of MexAB-OprM efflux pump. Another important point has to be noted that, all the novel mutation carrying *P*. *aeruginosa* were isolated from surgical ward and ICU of the hospital. However, the isolates showed a varying antibiogram pattern although all of them were multidrug resistant.

The present investigation warrants need for proteomics studies in order to verify the expression of a truncated protein at translational level. The antibiotic pressure in this particular region of the world might have played a significant role in selection of such mutation by bacteria. In the present study, we report a new mutation pattern in *mexR* gene that leads to overexpression of MexAB-OprM efflux pump in the clinical isolates of *P*. *aeruginosa*.

## Conclusion

This study highlighted emergence of a novel mutation which is probably associated with multi drug resistance. This opportunistic pathogen continues to threat the global health by accumulating novel mutations, complicating infection control management. Therefore, additional investigation and actions are needed to prevent or at least hold back the expansion and emergence of newer mutations in nosocomial pathogens which may compromise future treatment options. Molecular biological approach directing the investigation of the inducing agents for such mutations is crucial to overcome such critical infections. Further genomics and proteomics studies should also be taken up in order to understand the factors that contribute such mutation and overexpression of efflux pump system.

## Supporting Information

S1 Table Clinical details of *P*. *aeruginosa* isolates that demonstrated efflux pump activity phenotypically. (DOCX)
