# Correction: Premature Termination of MexR Leads to Overexpression of MexAB-OprM Efflux Pump in *Pseudomonas aeruginosa* in a Tertiary Referral Hospital in India

**DOI:** 10.1371/journal.pone.0151308

**Published:** 2016-03-11

**Authors:** Debarati Choudhury, Anamika Ghose, Debadatta Dhar Chanda, Anupam Das Talukdar, Manabendra Dutta Choudhury, Deepjyoti Paul, Anand Prakash Maurya, Atanu Chakravarty, Amitabha Bhattacharjee

There are errors in the second and eighth authors’ names. Their names should read: Anamika Ghose and Atanu Chakravarty. The corrected citation is: Choudhury D, Ghose A, Dhar Chanda D, Das Talukdar A, Dutta Choudhury M, Paul D, et al. (2016) Premature Termination of MexR Leads to Overexpression of MexAB-OprM Efflux Pump in *Pseudomonas aeruginosa* in a Tertiary Referral Hospital in India. PLoS ONE 11(2): e0149156. doi:10.1371/journal.pone.0149156.
