# Supplementary material for: Premature Termination of MexR Leads to Overexpression of MexAB-OprM Efflux Pump in Pseudomonas aeruginosa in a Tertiary Referral Hospital in India
Source: PLoS One. 2016 Feb 11;11(2):e0149156. doi: 10.1371/journal.pone.0149156 (PMC4750933; doi:10.1371/journal.pone.0149156)
Supplement: S1 Table — (DOCX) [file pone.0149156.s001.docx]

**S1 Table: Clinical details of *P. aeruginosa* isolates that demonstrated efflux pump activity phenotypically.**

| SL No. | Sample ID | Sex | Age (Years) | Ward/OPD | Type of clinical specimen |
| --- | --- | --- | --- | --- | --- |
|  | AM-D-64 | Male | 37 | ICU | Pus |
|  | AM-D-335 | Male | 53 | Surgery | Pus |
|  | AM-D-608 | Female | 56 | Surgery | Pus |
|  | AM-D-75 | Male | 31 | Medicine | Urine |
|  | AM-D-352 | Female | 29 | Ear Nose Throat | Oral swab |
|  | AM-D-529 | Male | 40 | Surgery | Pus |
|  | AM-D-326 | Female | 45 | Female burn Unit | Pus |
|  | AM-D-131 | Male | 35 | ICU | Pus |
|  | AM-D-173 | Female | 42 | Surgery | Urine |
|  | AM-D-146 | Female | 44 | Surgery | Pus |
|  | AM-D-67 | Female | 28 | ICU | Pus |
